# Takayasu Arteritis is Associated with Impaired Arterial Stiffness: A Meta-Analysis of Observational Studies

**DOI:** 10.31138/mjr.33.4.387

**Published:** 2022-12-31

**Authors:** Dimitrios Patoulias, Athina Dimosiari, Theodoros Michailidis

**Affiliations:** 1 Second Propaedeutic Department of Internal Medicine, Aristotle University of Thessaloniki, General Hospital “Hippokration”, Thessaloniki, Greece,; 2 Department of Internal Medicine, Interbalkan Medical Center, Thessaloniki, Greece; 3 Second Department of Internal Medicine, Aristotle University of Thessaloniki, General Hospital “Hippokration”, Thessaloniki, Greece

**Keywords:** Takayasu arteritis, arterial stiffness, cardiovascular disease

## Abstract

**Introduction/Objective:**

Takayasu arteritis, a large vessel vasculitis, is strongly associated with increased risk for cardiovascular and chronic kidney disease. Arterial stiffness represents an established prognostic marker of cardiovascular disease development in the general population. A few studies have assessed the effect of Takayasu arteritis on arterial stiffness indices. Herein, we sought to provide pooled effect estimates regarding the impact of Takayasu arteritis on arterial stiffness, by retrieving relevant, available observational studies.

**Methods:**

On 1^st^ May, 2022, we searched two major electronic databases and grey literature sources for relevant observational studies. We set as primary outcome the mean difference in carotid femoral PWV (cfPWV) between patients with Takayasu arteritis compared to controls.

**Results:**

Regarding the primary outcome, we pooled data from 3 studies in a total of 125 enrolled subjects, demonstrating that Takayasu arteritis is associated with a significant increase in cfPWV by 2.06 m/s (MD = 2.06, 95% CI; 1.29 – 2.83, I^2^ = 0%, p < 0.001), compared to controls.

**Conclusion:**

The present preliminary meta-analysis demonstrates the potential deleterious effects of Takayasu arteritis on arterial stiffness. Prognostic implications must be confirmed in larger, prospective studies.

## HIGHLIGHTS

- Patients with Takayasu arteritis experience an increased risk for all-cause mortality and cardiovascular morbidity and mortality.
- Arterial stiffness is an established prognostic marker of cardiovascular disease development in certain patient populations.
- Association between arterial stiffness and Takayasu arteritis has been addressed in a few observational studies. However, arterial stiffness assessment has not been widely adopted in Takayasu arteritis, in terms of primary of secondary prevention.
- In this meta-analysis, which is the first relevant in the literature, we demonstrate that Takayasu arteritis might be associated with a significant impairment in arterial stiffness indices, as measured by carotid-femoral pulse wave velocity. Prognostic implications of these results require further, well-designed, prospective observational studies.

## INTRODUCTION

Takayasu arteritis (pulseless disease), a large vessel vasculitis, predominantly affecting young Asian women, has long been considered as a rare disease.^1,2^ According to a recent meta-analysis of observational studies, incidence rate of Takayasu arteritis is 1.11 per million person-years, significantly more common among women.^3^ However, there is evidence of significant geographical disparities concerning the prevalence of disease.^4,5^

Patients with Takayasu arteritis feature a significantly increased risk for all-cause mortality, along with an increased risk for cardiovascular disease and chronic kidney disease.^6^ Pulmonary hypertension seems to be an additional burden for patients suffering from Takayasu arteritis.^7^ Therefore, there is a need for prognostic markers of cardiovascular disease occurrence for this sensitive population.

Arterial stiffness is an established prognostic marker of cardiovascular disease in the general population.^8^ Increase in pulse wave velocity (PWV), the gold standard of arterial stiffness, by 1 m/s is associated with a significant increase in the risk for major adverse cardiovascular events, cardiovascular mortality and all-cause mortality by 14%, 15%, and 15%, respectively.^8^ Thus, it seems that PWV might be a useful prognostic tool for cardiovascular disease prevention in patients with Takayasu arteritis.

Therefore, we sought to determine the impact of Takayasu arteritis on arterial stiffness indices, compared to controls, by performing the first in the literature systematic review and meta-analysis of relevant observational studies.

## METHODS

This systematic review and meta-analysis is conducted in accordance with the PRISMA (Preferred Reporting Items for Systematic reviews and Meta-Analyses) guidelines^9^ and the Meta-analysis Of Observational Studies in Epidemiology (MOOSE) guidelines.^10^

### Eligibility

We searched for observational studies enrolling adult patients with Takayasu arteritis, compared to healthy controls, assessing major arterial stiffness indices. We excluded case series, case reports, former meta-analyses (if any), Editorial and opinion papers and narrative reviews.

### Search

We searched on 1^st^ May 2022 the PubMed and Scopus databases, utilising the search terms “Takayasu arteritis”, “arterial stiffness”, “pulse wave velocity” and “PWV”. Grey literature sources, namely conference proceedings, were also searched. We did not impose any filter regarding sample size, study setting, or publication language.

### Outcomes

We set as primary outcome the mean difference in carotid femoral PWV (cfPWV) between patients with Takayasu arteritis compared to selected controls.

### Data extraction

After deduplication, two independent reviewers (D.P., A.D.) screened all records at title and abstract level and then assessed the full text of eligible records. Any disagreements were resolved by consultation of a third reviewer (T.D.).

Three independent reviewers (D.P., A.D. and T.M.) extracted the data from the eligible reports. Extracted information were: first author, year of study conduction, study setting, study sample size, country of origin, measurement method of PWV, measured PWV in each arm, mean age of participants, male to female ratio, major comorbidities and concomitant treatment of interest.

### Statistical analysis

Differences were calculated with the use of mean difference (MD), with 95% confidence interval (CI), after implementation of the Mantel-Haenszel (M-H) random effects formula. Statistical heterogeneity among studies was assessed by using I^2^ statistics. I^2^ ranging between 0 and 40% is considered as low, I^2^ ranging between 50% to 90% may represent substantial heterogeneity and I^2^ ranging between 75% to 100% may be indicative of considerable heterogeneity. All analyses were performed at the 0.05 significance level, with the RevMan 5.3. software.

### Quality assessment

Two independent reviewers (D.P. and A.D.) assessed the quality of the included observational studies, by using the Newcastle-Ottawa Scale (NOS).^11^ Studies were judged on 3 broad perspectives: selection of the study groups; comparability of the groups; and ascertainment of either the exposure or outcomes of interest. A maximum of 4 stars for selection, 2 stars for comparability, and 3 stars for outcome can be awarded to any individual study, for a maximum of 9 stars per study. Discrepancies between reviewers were solved by discussion, consensus, or arbitration by a third senior reviewer (T.D.).

## RESULTS

**Figure 1.**
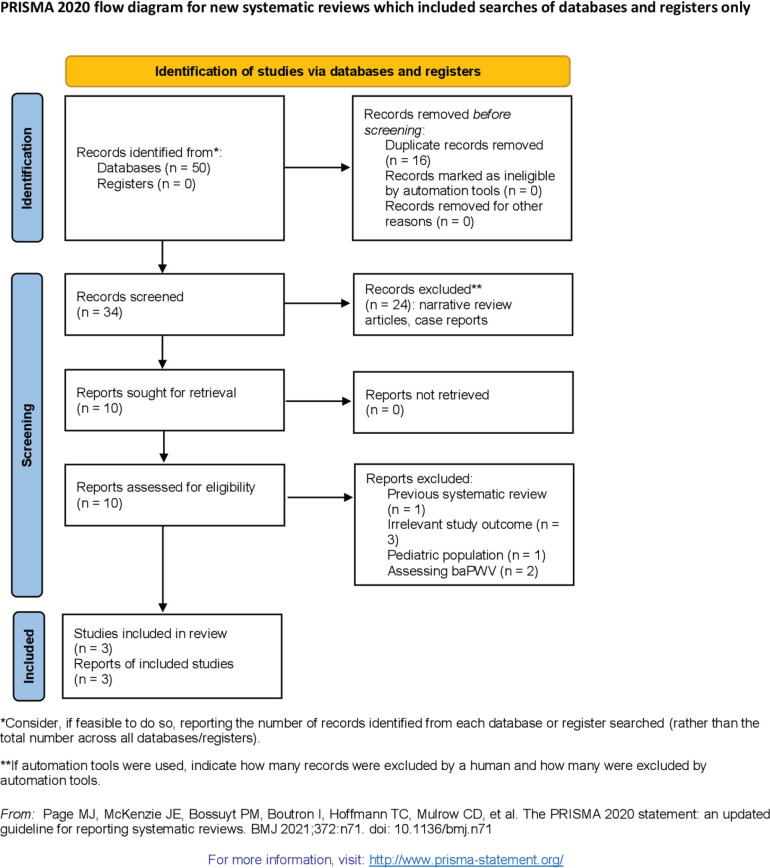
PRISMA flow diagram depicting the study selection process.

**Figure 2.**
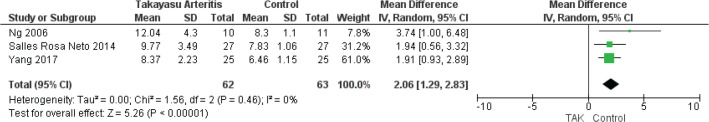
Mean difference in cfPWV between patients with Takayasu arteritis and controls.

As shown in the corresponding PRISMA flow diagram (**Figure 1**), our search strategy retrieved 50 results in total. After deduplication, we initially screened 34 records at title and abstract level. Finally, we assessed 10 records in full text. Three of them were evaluated as eligible for inclusion in our qualitative and quantitative synthesis.^12–14^ One observational study was excluded, as it was performed in a paediatric population.^17^ Two studies were excluded, since they assessed branchial ankle PWV (baPWV).^15,16^ A detailed description of participants’ baseline characteristics is provided in **Table 1**. Regarding the primary outcome, we pooled data from 3 studies in a total of 125 enrolled subjects.^14–16^ We demonstrated that Takayasu arteritis is associated with a significant increase in cfPWV by 2.06 m/s (MD = 2.06, 95% CI; 1.29 – 2.83, I^2^ = 0%, p < 0.001), compared to controls, as shown in **Figure 2**. Notably, none of the 3 eligible studies documented a significant correlation between inflammatory, laboratory markers, mainly C-reactive protein (CRP) and erythrocyte sedimentation rate (ESR), and cfPWV.

**Table 1.** Participants’ baseline characteristics of interest.

**Studies**	**Number of patients (n)**	**Age (years)**	**Male/female (n)**	**Body mass index (kg/m^2^)**	**Hypertension (%)**	**Diabetes mellitus (%)**	**Dyslipidemia (%)**	**Cardiovascular disease (%)**	**Correlatin of cfPWV with inflammatory markers**
**Ng (2006)**	21	TAK: 41.0 ± 12.5 C: 32.4 ± 5.5	TAK: 0/10 C: 0/11	TAK: 26.3 ± 3.1 C: 22.2 ± 1.9	Not reported	Not reported	Not reported	Not reported	Non-significant
**Salles Rosa Neto (2014)**	54	TAK: 32.4 ± 8.3 C: 33.9 ± 10.1	TAK: 0/27 C: 0/27	TAK: 22.3 ± 2.6 C: 23.7 ± 3	TAK: 66.7% C: Not reported	Not reported	TAK: 22.2% C: Not reported	Not reported	Non-significant
**Yang (2017)**	50	TAK: 28.3 ± 6.2 C: 27.1 ±4.2	TAK: 0/25 C: 0/25	TAK: 20.7 ± 2.9 C: 20.9 ± 3.0	0%	0%	0%	0%	Non-significant

* TAK: Takayasu arteritis patients; C: controls

† Data is presented as mean ± standard deviation, absolute numbers (n) or relative frequencies (%), unless otherwise stated.

All trials were graded as of good quality, according to the NOS scale (**Supplementary Table 1**). No statistical heterogeneity was shown for the primary efficacy outcome.

## DISCUSSION

In the present meta-analysis, we have documented that Takayasu arteritis might be associated with a significant increase in cfPWV, compared to controls, indicating that arterial stiffness might represent a valuable cardiovascular risk marker for affected subjects.^12–14^ Of note, both studies assessing the impact of Takayasu arteritis on baPWV, also showed significantly greater baPWV values in patients compared to controls.^15,16^

In a former observational study by He and colleagues, it was demonstrated that baPWV was an independent predictor of major adverse cardiovascular events (MACEs) among patients with Takayasu arteritis, strongly correlated with age, blood pressure, angiographic stage and renal involvement during disease course.^18^ Another observational study by Wang and Dang documented the strong association between baPWV and MACEs in patients with Takayasu arteritis and established coronary artery disease, underlining the prognostic value of arterial stiffness in terms of secondary prevention.^15^ Of course, cfPWV represents the gold-standard for the assessment of arterial stiffening^19^; however, no studies have assessed the association between cfPWV and cardiovascular disease occurrence in patients with Takayasu arteritis so far. In addition, further evidence is required in order to confirm or not whether current treatment options for refractory Takayasu arteritis, such as tumour necrosis factor-α (TNF-α) antagonists, tocilizumab and tofacitinib,^20–22^ exert any cardio-protective effect in affected subjects, and if arterial stiffness mediates at some extent the observed cardio-protection.

Of course, current evidence is insufficient to document whether there is greater increase in cfPWV among patients with active disease compared to those being in remission. Yang et al.^12^ enrolled hospitalized patients with Takayasu arteritis, however they did not document any significant association between cfPWV and either CRP (p = 0.11) or ESR (p = 0.13). Salles Rosa Neto and colleagues^13^ recruited 27 subjects with Takayasu arteritis, of whom 15 had active disease and 12 were on remission; however, no significant difference between the two subgroups of patients was shown by the researchers (p = 0.83), while no significant association between cfPWV and CRP (p = 0.71) or ESR (p = 0.17) was found. In their trial, Ng et al.^14^ failed to show a significant correlation between cfPWV values and disease activity (p = 0.19), as assessed with the use of Kerr’s criteria, while no correlation between arterial stiffness measurements and CRP (p = 0.23) or ESR (p = 0.27) was shown. Although inflammation, even at a subclinical level, is associated with increased arterial stiffness and overall cardiovascular risk,^23–25^ preliminary data from some patients’ populations have not detected a clinically meaningful association between baseline levels of inflammatory markers and changes in metrics of arterial stiffness over time and during disease course. Besides the reasonable arising question concerning the association between arterial stiffness indices and systemic or vascular inflammation, another point that requires further research to be answered is whether Takayasu arteritis, as a vasculitis affecting large vessels leading to stenoses, is a priori associated with increased arterial stiffness. Limited and old evidence has suggested an association between severity of angiographic involvement in patients with suspected coronary artery disease and vascular stiffness.^26^ However, no such data exist for patients with rheumatic diseases. Unfortunately, none of the eligible studies provided relevant information regarding the angiographic involvement due to disease and its association with cfPWV; however, it should be highlighted that in the trial by Salles Rosa Neto et al.^13^ patients with Takayasu arteritis that had undergone vascular surgery due to disease had significant greater cfWPV measurements compared to those that did not have a history of vascular surgery (p = 0.03). This might be implicating of a significant association between stenotic disease progress and cfPWV, although no such data exist so far.

We consider as main limitations of the present analysis the limited number of eligible observational studies and the small sample size of them. In addition, absence of individual participant data did not permit us to perform subgroup analyses of the assessed arterial stiffness indices according to the prior history of cardiovascular comorbidities or cardiovascular disease, or baseline medication of specific interest, with established beneficial effect on arterial stiffness indices, such as renin-angiotensin-aldosterone blockers, or the baseline immunosuppressive treatment. In general, included studies did not report data regarding the presence of cardiovascular risk factors at baseline, and whether a multivariate analysis was performed, in order to assess the true impact of Takayasu arteritis on cfPWV. Finally, we have not registered our protocol at a publicly available repository, constituting an additional limitation.

To sum up, the results of the present meta-analysis, despite being preliminary, provide some data regarding the deleterious effects of Takayasu arteritis progression on arterial stiffness. Prognostic implications of this observation must be further confirmed in larger, prospective studies.

## Figures and Tables

**Supplementary Table 1.** Newcastle-Ottawa Scale for the assessment of the quality of the eligible observational studies.

**Studies**	**Selection**	**Comparability**	**Exposure**	**Overall grade**
**Grotenhuis (2019)**	***	**	***	Good quality
**Ng (2006)**	***	**	**	Good quality
**Salles Rosa Neto (2014)**	***	**	***	Good quality
**Yang (2017)**	***	**	***	Good quality
**Wang (2015)**	***	**	***	Good quality
**Wang (2020)**	***	**	**	Good quality
